# A Comparison of Prices Paid by London Hospitals for Their Food Supplies.—II
*The first "comparison" appeared last week.


**Published:** 1912-04-13

**Authors:** 


### INSTITUTIONAL HOUSEKEEPING.

### A Comparison of Prices Paid by London Hospitals for their Food Supplies.—II.*

The analysis of prices given below exhibits extraordinary variety. While one hospital apparently feeds all inmates on the finest Vienna flour and bread, a luxury presenting no additional food value, another is revealed using a "very inferior quality" of both. In one institution the inmates are being fed on "chicken rice," while in another some contractor is making splendid profits out of the ignorance displayed as to the market price of ice. Are these things accounted for by the following note appended by the authorities of the King's Fund to their report?

"The circumstances in which goods are purchased, the quantities and qualities, the date and length of contract, and conditions of delivery would have to be taken into consideration before assuming that the lowest prices paid by a single hospital in any year were obtainable in every institution."

Making every possible allowance for such circumstances, can one believe that the variations indicated below in the minimum cost of common necessaries are justifiable?

*Flour.*—Per sack (280 lb.). Highest price, £2 6s. 8d.; lowest price, £1 5s. 6¼d. We presume that the article costing 46s. 8d. per sack is first-class Hungarian, which is not used for ordinary household purposes except when blended with other flours, and that being the case the quotation is somewhat misleading. For ordinary purposes certainly not more than 30s. per sack should be paid for household flour bought by contract, and at this price good English flour can generally be bought. On the other hand, 25s. 6¼d. is exceedingly low for flour of good quality, but a low-grade American article, which is wholesome if not of particularly good colour and flavour, can generally be obtained for about this figure.

*Bread.*—Highest price, 8d.; lowest price, 4d. per 4 lb. The prices of this commodity are subject to the same conditions as those of flour. "Vienna" bread made with Hungarian, or a mixture of Hungarian and English flour, is worth 8d. per 4 lb., whilst 4d. is about the lowest price at which household bread of any quality could be obtained. As in the case of flour the quality and colour of the cheapest article would be very inferior, and a more satisfactory result would probably be obtained by the payment of a slightly higher price, and a guarantee from the baker regarding the blend of flour used in the manufacture.

*Tea.*—The quotations do not show any great extravagance, 1s. 8d. being a moderate price for tea of good quality; for 11½d. per lb., which was the lowest price paid, only the sweepings of the market could be got.

*Sugar.*—Highest price, £1 4s. 6d.; lowest price, 10s. per cwt. There are so many grades and kinds of this article that one does not gather much from the prices quoted, but presuming the minimum to represent the cheapest cooking sugar, and the maximum the finest loaf, we should say that the first is very well bought indeed, and the second quite worth the money paid; in regard to the latter, however, we should very much doubt whether a guaranteed pure edible sugar could have been purchased at anything like such a low price; we should have expected the minimum to have been about 14s.

*Rice.*—Highest price, £1 2s.; lowest price, 7s. per cwt. Here again the minimum price is a good deal lower than we should expect; anything purchased at less than 10s. or 11s. per cwt. is unfit for culinary purposes, but "chicken rice," *i.e.* grain broken and of bad colour, is obtainable for less money; 20s. is not an extravagant price for good Patna or Java, whilst anything from 30s. upwards would be paid for genuine Carolina. The last-named is not generally used in hospitals.

*Potatoes.*—Highest price, 9s.; lowest price, 3s. 3d. per cwt. We are unable to suggest any reason for the payment of 9s. per cwt. for potatoes, as Scotch Dunbars, acknowledged to be the very best, are obtainable from any good greengrocer at 6s. 6d. or 7s., whilst good Blacklanders can generally be bought at 5s. We should, however, consider it poor economy to buy potatoes at 3s. 3d. per cwt., as anything like a reliable article could not possibly be supplied at that price.

*Ice.*—Highest price, 4s.; lowest price, $11\frac{1}{10}$d. per cwt. 4s. per cwt. is an enormous price for this article, which is always obtainable upon contract in the Metropolis at something less than 1s. 6d. If this were the "carriage paid" quotation to some institution situated in a remote part of the country it might be comprehensible, but we can imagine no reason for such a high price in London. The institution quoting at $11\frac{1}{10}$d. must either have discovered a very favourable market, or be making their own supply, as is done at the Middlesex Hospital and several others.

* The first "comparison" appeared last week.

### HOUSEKEEPING QUERIES.

Under this heading questions are dealt with relating to cookery and household recipes, the duties of the domestic staff, estimates of quantities required for given numbers, and the cost of diet for patients and staff. Arrangements have been made to deal with samples submitted for examination and report on them.

Home-made Baking-powder ("M. B.").—It is distinctly cheaper to prepare baking-powder at home. There are many recipes for it, but you will find the following excellent: Pass three times through a hair or fine-wire sieve ½ lb. of cream of tartar, ¼ lb. bicarbonate of soda, ¼ lb. ground rice. A pound of a similar preparation would cost about two shillings; it can be made at home for about ninepence halfpenny.

